# Mediterranean-like Dietary Pattern and Serum Phosphorus in Hemodialysis: Beyond Food Phosphorus Content

**DOI:** 10.3390/nu18172877

**Published:** 2026-09-02

**Authors:** Ana Valente, Inês Pastor Neto, Joana Jesus, Cristina Garagarza, Aníbal Ferreira

**Affiliations:** 1 Nutrition Department, Nephrocare Portugal S.A., 1750-233 Lisbon, Portugal; ana.valente@freseniusmedicalcare.com (A.V.); ines.neto@freseniusmedicalcare.com (I.P.N.); joana.jesus@freseniusmedicalcare.com (J.J.); 2 Nutrition Laboratory, Faculty of Medicine, Lisbon University, 1649-028 Lisbon, Portugal; 3 Dialysis Unit Carregado, Nephrology Department, Nephrocare Portugal S.A., 2580-588 Carregado, Portugal; anibalferreira@netcabo.pt; 4 Faculty of Medical Sciences, NOVA Medical School, 1169-056 Lisbon, Portugal

**Keywords:** serum phosphorus, hemodialysis, mediterranean-like dietary pattern, western-like dietary pattern

## Abstract

Background: Disturbances in mineral and bone metabolism are common in hemodialysis (HD) patients, increasing the risk of hyperphosphatemia. Dietary phosphorus restriction remains a cornerstone of management, often leading to stringent dietary recommendations. This study aimed to explore whether adopting a Mediterranean-like dietary pattern (Med-LDP), compared to a Western-like dietary pattern (West-LDP) was associated with serum phosphorus levels. Methods: This cross-sectional multicenter study included 564 HD patients from 37 dialysis centers. Dietary intake was assessed using a food frequency questionnaire, and dietary patterns were identified through principal component analysis based on 22 food groups. Associations between dietary patterns, phosphorus intake, and serum phosphorus levels were evaluated using correlations tests and linear regression analyses adjusted for demographic, clinical, nutritional, and dialysis-related factors. Results: Two different dietary patterns were identified: a “Mediterranean-like” pattern characterized by a higher intake of vegetables, legumes, fruit, starchy products, fish, olive oil, eggs, bakery products, and alcohol; and a “Western-like” characterized by a higher intake of soft drinks, red meat, fried and salted foods, fast food, and caffeine. Despite significantly higher intakes of energy, protein, carbohydrates, phosphorus, potassium, and fiber, patients following the Med-LDP exhibited lower serum phosphorus levels, calcium–phosphorus product, and PTHi concentrations. No differences were observed in serum potassium levels. The correlation between phosphorus intake and serum phosphorus was weak and not statistically significant. The West-LDP was positively associated with serum phosphorus levels and remained an independent predictor after adjustment for potential confounders. However, after further adjustment for the prescribed dosages of phosphate binders, vitamin D supplementation and analogs, as well as calcimimetics, the association was no longer statistically significant. Conclusions: HD patients following a Med-LDP exhibited lower serum phosphorus levels despite a higher phosphorus intake. These findings suggest that the overall dietary pattern may be a more important determinant of phosphorus homeostasis than total phosphorus intake alone.

## 1. Introduction

Hyperphosphatemia, defined as elevated serum phosphorus concentrations, is one of the most common metabolic disturbances in patients undergoing maintenance hemodialysis (HD), affecting more than half of this population despite advances in dialysis techniques, phosphate binders, and dietary management strategies [1].

Several factors influence phosphorus homeostasis, including dietary phosphorus absorption, renal phosphate reabsorption, and the balance between bone formation and resorption [2]. As chronic kidney disease (CKD) progresses, these mechanisms become impaired, leading to vitamin D deficiency, reduced calcium absorption, and diminished renal phosphorus excretion, thereby exacerbating phosphorus and calcium imbalances. This also contributes to secondary hyperparathyroidism, stimulating bone turnover and resorption and further increasing phosphorus levels [2,3]. Consequently, hyperphosphatemia is a key component of CKD-mineral and bone disorder (CKD-MBD) and is associated with increased morbidity as it contributes to vascular calcification, bone demineralization, and cell signaling and apoptosis dysregulation, thereby increasing the risk of fractures and cardiovascular (CV) events, as well as overall and CV-related mortality [2,3]. A recent systematic review found that every 1 mg/dL increase in serum phosphorus was associated with an 18% increase in the risk of mortality [4].

The management of hyperphosphatemia is based on three fundamental pillars: dietary and lifestyle modifications, dialysis, and pharmacological treatment (including phosphate binders, active vitamin D and vitamin D analogs, and/or calcimimetics) [2]. Dietary phosphorus restriction has long been a key strategy, with older guidelines suggesting 800–1000 mg/day [5]. However, recent evidence suggests that phosphorus management should extend beyond total intake and consider additional nutritional factors affecting phosphorus absorption, such as the food source and matrix, the relative amounts of dietary calcium, phosphate, and vitamin D sterols, bile acid concentration, and cooking methods [5,6]. This perspective has shaped the updated KDOQI guidelines, which recommend not only adjusting dietary phosphorus intake to maintain normal serum phosphate levels but also considering the bioavailability of different phosphorus sources [5]. Animal-derived and highly processed foods containing phosphate additives have significantly higher bioavailability than plant-based sources [2,6]. Consequently, emerging literature and recent guidelines advocate reducing the consumption of ultra-processed foods with high phosphate additive content, as this may be associated with improved phosphate control [6]. There is growing recognition that assessing the overall dietary pattern may be more appropriate than focusing solely on total phosphorus intake. A Western diet—characterized by a high intake of red meat, animal fat, and highly processed foods preserved with phosphate and sodium, alongside a low intake of fresh fruits and vegetables—is estimated to provide between 1000 and 1500 mg of phosphorus per day, with 10–50% coming from food additives [6]. In contrast, plant-based diets may supply similar phosphorus intake, but with lower bioavailability [6,7]. These diets promote the consumption of vegetables, fruits, legumes, nuts, whole grains, and unsaturated vegetable oils while allowing the moderate consumption of animal products and limiting refined carbohydrates, sweets, and ultra-processed foods.

Furthermore, plant-based diets, including the Mediterranean diet and the Dietary Approaches to Stop Hypertension (DASH) diets, have been associated, across diverse populations, with a reduced risk of age-related cognitive decline, dementia, Alzheimer’s disease, cardiovascular disease, and all-cause mortality [8,9,10,11,12,13].

Despite this, there is still no evidence supporting the recommendation of a specific dietary pattern to control phosphorus levels in HD patients, and few studies have focused on dietary patterns in this population. This raises a key question: could Western diets increase the risk of hyperphosphatemia, while plant-based diets—widely linked to better health outcomes—be associated with better maintenance of phosphorus balance in HD patients? Therefore, this study aimed to explore whether adopting a Mediterranean-like dietary pattern (Med-LDP), compared with a Western-like dietary pattern (West-LDP) was associated with lower serum phosphorus levels.

## 2. Materials and Methods

### 2.1. Study Design and Subjects

This was a cross-sectional, multicenter, observational study that included 564 patients undergoing maintenance HD at 37 dialysis centers in Portugal.

Patients aged ≥ 18 years who had been receiving thrice-weekly, 4 h, in-center HD using online hemodiafiltration for ≥15 months and who agreed to participate and provided written informed consent were included in the study. The minimum HD vintage of 15 months was defined considering that the Food Frequency Questionnaire (FFQ) assesses dietary habits during the 12 months preceding its administration, in addition to the usual 3-months period defined in most HD studies, which is considered as an adaptation period to treatment and dietary recommendations. The exclusion criteria were: limited understanding of Portuguese, severe neurological or mental disorders, active neoplastic disease, major amputation (lower/upper extremities), enteral or parenteral feeding, severe alcohol or drug addiction, hepatitis C with viral replication, liver disease, and treatment with immunosuppressive agents or corticosteroids.

Among the 4600 patients undergoing HD across 37 dialysis centers, 600 patients meeting the eligibility criteria were randomly selected in equal proportions from each center. Of these, 18 patients (3%) declined to participate, resulting in a study population of 582 patients. Among the enrolled participants, 564 had available pre-dialysis serum phosphorus measurements and were therefore included in the final study population.

Patients underwent post-dilution online hemodiafiltration using high-flux polysulfone membranes (Helixone^®^; Fresenius Medical Care^®^, Bad Homburg, Germany) and ultrapure water that met the requirements of the International Organization for Standardization (ISO) 13959:2009 standard for water used in hemodialysis and related therapies.

### 2.2. Data Analysis

Demographic, anthropometric, body composition, biochemical parameters as well as dialysis treatment data were collected from the patients’ clinical records during the same month in which the FFQ was administered.

### 2.3. Anthropometric and Body Composition Analysis

Body composition was assessed using bioimpedance spectroscopy with the Body Composition Monitor (BCM) (version 3.3, Fresenius Medical Care^®^ Deutschland GmbH, Bad Homburg, Germany) which has been validated against the gold standard reference methods in several studies involving more than 500 patients and healthy controls [14,15,16,17]. The measurements had a quality index greater than 95%. This evaluation was performed with the patient lying supine, approximately 30 min before the midweek HD sessions, with four conventional electrodes (Fresenius Medical Care): two placed on the hand and two on the foot contralateral to the vascular access. To ensure measurement quality, only measurements with a quality indicator above 90% were accepted. This quality indicator is provided by the BCM, which performs measurements at 50 frequencies ranging from 5 to 1000 kHz. The body composition analysis included the lean tissue index (LTI), fat tissue index (FTI), body cell mass index (BCMI), fat mass (%), lean tissue mass (%), intracellular water (l), extracellular water (l), and relative overhydration (%) (pre-dialysis overhydration/extracellular water [OH/ECW]).

### 2.4. Dietary Intake

Dietary intake was assessed using a semi-quantitative FFQ, developed and validated for the Portuguese population [18,19]. The questionnaire was administered by a registered dietitian in a face-to-face interview while the patient was undergoing HD treatment. The FFQ included 95 food items, a section with predetermined average portion sizes, and nine intake-frequency categories ranging from “never or less than once a month” to “six or more times a day”. Patients were asked to describe their dietary habits during the previous 12 months, specifically the frequency of consumption and the mean portion size of each food item. A booklet containing 131 color photos illustrating food servings was used as a visual aid.

Dietary intake was then estimated by multiplying the consumption frequency of each item by its respective portion size (in grams), and a seasonal factor variation factor. Information on daily intake of macro- and micronutrients was extracted from this questionnaire using Food Processor Plus software (version 11.7.217; ESHA Research, Salem, OR, USA), which contains nutritional data from the United States Department of Agriculture. The database was supplemented with information on nutrient content of Portuguese foods obtained from the Portuguese food composition table [20]. Before statistical analysis, all food items with a mean intake of ≤5 g/day were excluded using an investigator-defined operational threshold, consistent with our previously published methodology [21], to reduce the number of variables representing foods with very low average consumption in the study population.

Twenty-two food groups were created based on similar nutritional characteristics: dairy products; eggs; white meat; red meat; fish; white bread, rice, pasta and potatoes; whole grain bread; vegetables; fruit; vegetable soup; chicken soup and Portuguese green soup; legumes; nuts; olives and olive oil; oils; fried and savory snacks; fast food; bakery products; alcoholic beverages; caffeinated drinks; barley drinks; and soft drinks. Each food group was adjusted for total energy intake and used to define dietary patterns in this population. The patterns were named according to the food groups with the highest factor loadings.

### 2.5. Laboratory Variables

The biochemical parameters of the overall population were assessed and compared between the dietary pattern groups, including sodium, potassium, phosphorus, calcium, calcium/phosphorus product (Ca/P), intact parathyroid hormone (PTHi), normalized protein catabolic rate (nPCR), albumin, ferritin and hemoglobin. Blood samples used for biochemical analysis were collected before the midweek HD session.

Serum sodium and potassium concentrations were determined by indirect potentiometry, whereas phosphorus and calcium concentrations were assessed using the UV molybdate and o-cresolphthalein colorimetric methods, respectively. iPTH and ferritin concentrations were quantified by chemiluminescent immunoassay (CLIA). Hemoglobin was measured by spectrophotometry in whole blood, and serum albumin was determined using the bromocresol green method. All laboratory parameters were measured using identical methods across the different laboratories.

### 2.6. Statistical Analysis

Dietary patterns were derived from principal component analysis (PCA) based on 22 food groups. PCA with varimax rotation and Kaiser normalization was applied to improve the interpretability of the factors. The number of dietary patterns retained was determined based on eigenvalues > 1.5 (which indicate the total variance explained by a given factor), as well as the scree plot examination, and the interpretability of the derived patterns. Adherence to each dietary pattern was then determined using the factor scores generated by the PCA, and participants were assigned to the dietary pattern for which they had the highest factor score. This approach was chosen because the aim of the study was to characterize the predominant dietary patterns within the study population and compare the clinical characteristics of participants according to their predominant dietary profile.

Data distribution was evaluated using the Kolmogorov–Smirnov test. Normally distributed continuous variables were expressed as the mean (standard deviation [SD]), whereas median (interquartile range [IQR]) was used for non-normally distributed continuous variables. Categorical variables were presented as frequencies and percentages. Pearson’s chi-squared test was used to analyze categorical variables. Differences between groups were evaluated using the independent samples *t*-test for normally distributed variables, and the Mann–Whitney test for non-normally distributed variables. To complement *p*-values, effect sizes and their 95% confidence intervals (95% CIs) were reported. Hedges’ g was used for independent samples *t*-tests, Hodges–Lehmann estimates of median differences were used for the Mann–Whitney test, and odds ratios (ORs) and Cramer’s V were used for categorical variables with two and more than two categories, respectively. Confidence intervals for Cramer’s V were estimated using bias-corrected and accelerated (BCa) bootstrap methods. Spearman’s correlation tests, and linear regression analyses were performed to evaluate the associations of dietary phosphorus intake, phosphorus/protein ratio, and dietary patterns with serum phosphorus levels. The multivariate analyses was adjusted for age, sex, presence of diabetes mellitus, energy intake, phosphorus/protein intake ratio, dialysis adequacy, HD vintage, vascular access, PTHi, albumin, residual diuresis (patient-reported), and the prescribed weekly doses of phosphorus binders (calcium acetate/magnesium carbonate, sevelamer carbonate, and sucroferric oxyhydroxide), calcimimetics (cinacalcet), and vitamin D supplementation and analogs (alfacalcidol, paricalcitol, and calcitriol). All statistical analyses were performed using the IBM SPSS Statistics software (version 29.0; IBM Corp., Armonk, NY, USA), and a *p*-value < 0.05 was considered statistically significant.

## 3. Results

Two different dietary patterns were identified (Table 1): a Med-LDP characterized by a higher intake of vegetables (109 ± 99 g/day), legumes (22 ± 30 g/day), fruit (314 ± 217 g/day), starchy products (278 ± 190 g/day), fish (69 ± 46 g/day), olive oil [14 ± 15 g/day), eggs (22 ± 23 g/day), bakery products (56 ± 72 g/day), and alcohol (95 ± 155 mL/day); and a West-LDP characterized by higher intake of soft drinks (70 ± 121 mL/day), red meat (71 ± 188 g/day), fried and savory snacks (13 ± 18 g/day), fast food (7 ± 14 g/day), and caffeinated drinks (107 ± 113 mL/day) (Table 2).

**Figure 1:**
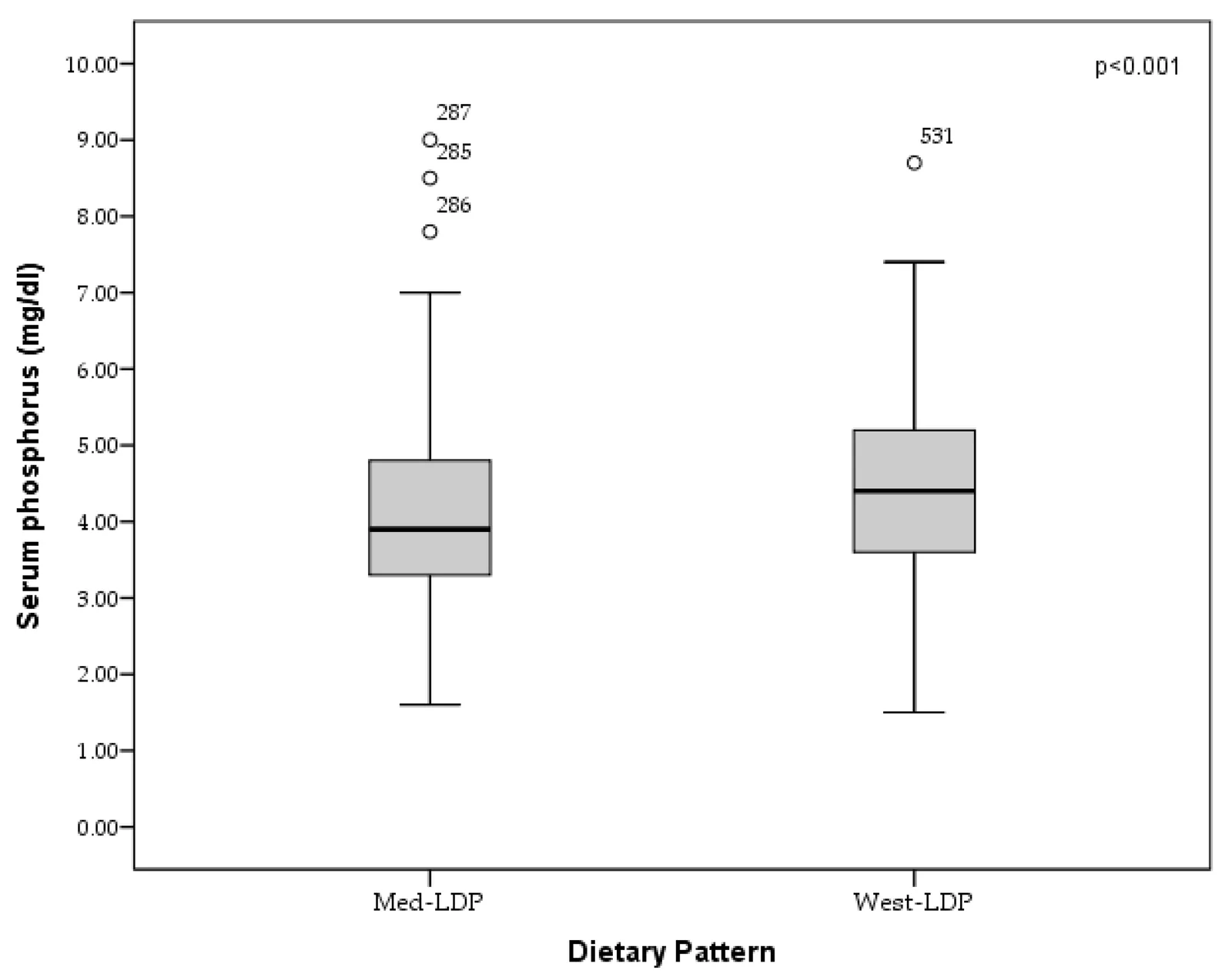
Distribution of serum phosphorus concentrations according to dietary pattern.

The median age of the overall population was 71 years (IQR: 61–78), with a median HD vintage of 65 months (IQR: 43–105). Male participants accounted for 41.3% of the cohort, while 31.4% had diabetes mellitus (DM). Regarding vascular access for hemodialysis, 83% of patients used arteriovenous fistulas (Table 3). Median serum phosphorus differed between the dietary patterns, with the patients following a West-LDP having higher serum phosphorus levels (Figure 1). The characteristics of the overall population, as well as differences in demographic, body composition, biochemical, and dialysis-related parameters between patients following a Med-LDP and those following a West-LDP, are presented in Table 3. Regarding phosphate binder prescriptions, 26.6%, 3.55% and 17.38% of the overall population were prescribed calcium acetate/magnesium carbonate, sucroferric oxyhydroxide, and sevelamer carbonate, respectively. Only sevelamer carbonate prescription differed significantly between dietary patterns (*p* = 0.001), with 33 patients (12.04%) in the Med-LDP and 65 (22.41%) in the West-LDP group receiving this phosphate binder.

Despite the higher intakes of protein, carbohydrates, calories, fat, phosphorus, and potassium in the Med-LDP group, serum phosphorus levels, nPCR, Ca/P product, and PTHi were significantly lower (*p* < 0.05). Patients in the Med-LDP group also had significantly higher intakes of fiber, vitamins (A, B complex, C, D, E, K), and minerals (including calcium, iron, magnesium, manganese, phosphorus, potassium, sodium, zinc, and selenium) (Table 4). However, no statistically significant differences were observed between the two groups in serum of potassium, ferritin, hemoglobin, calcium, and sodium (*p* > 0.05) (Table 3).

**Figure 2:**
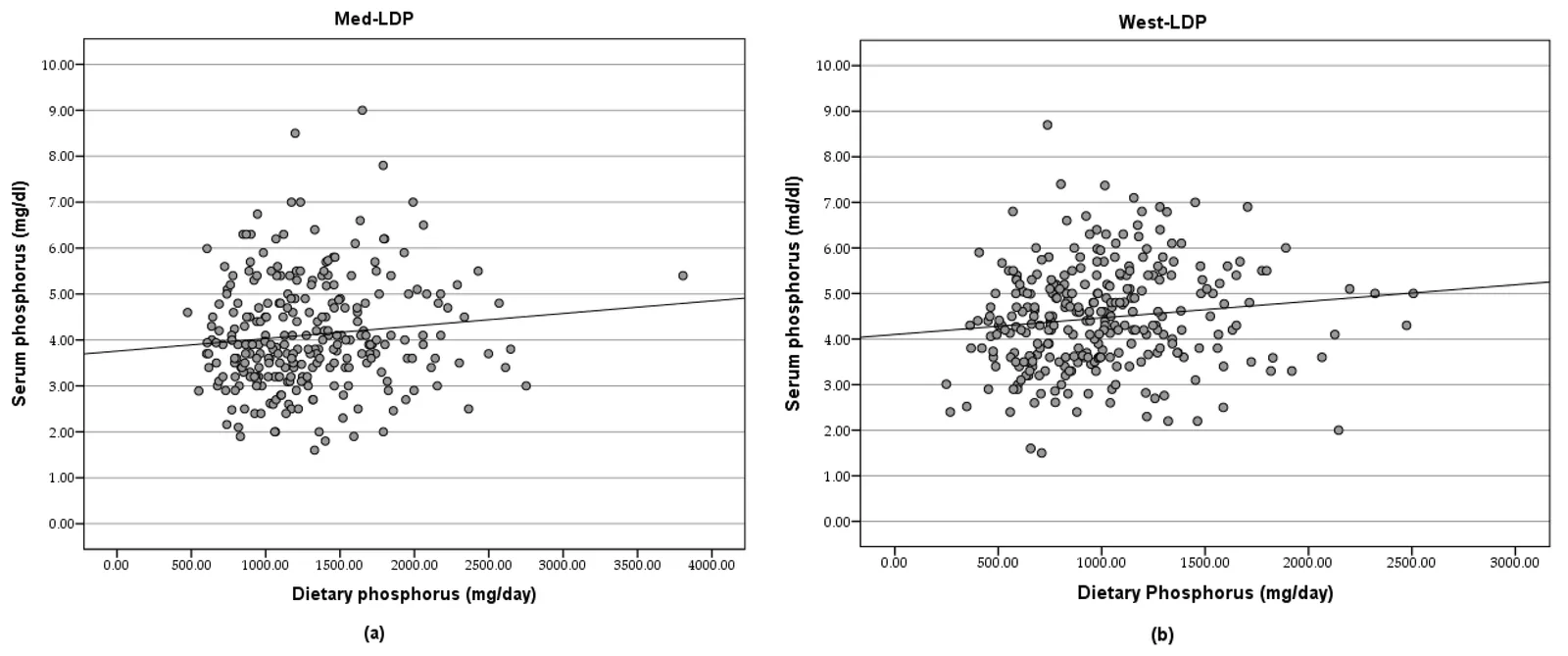
Correlation between serum phosphorus and dietary phosphorus intake, according to study group. (**a**) Med-LDP: ρ = 0.114, *p* = 0.059. (**b**) West-LDP: ρ = 0.0171, *p* = 0.004.

**Figure 3:**
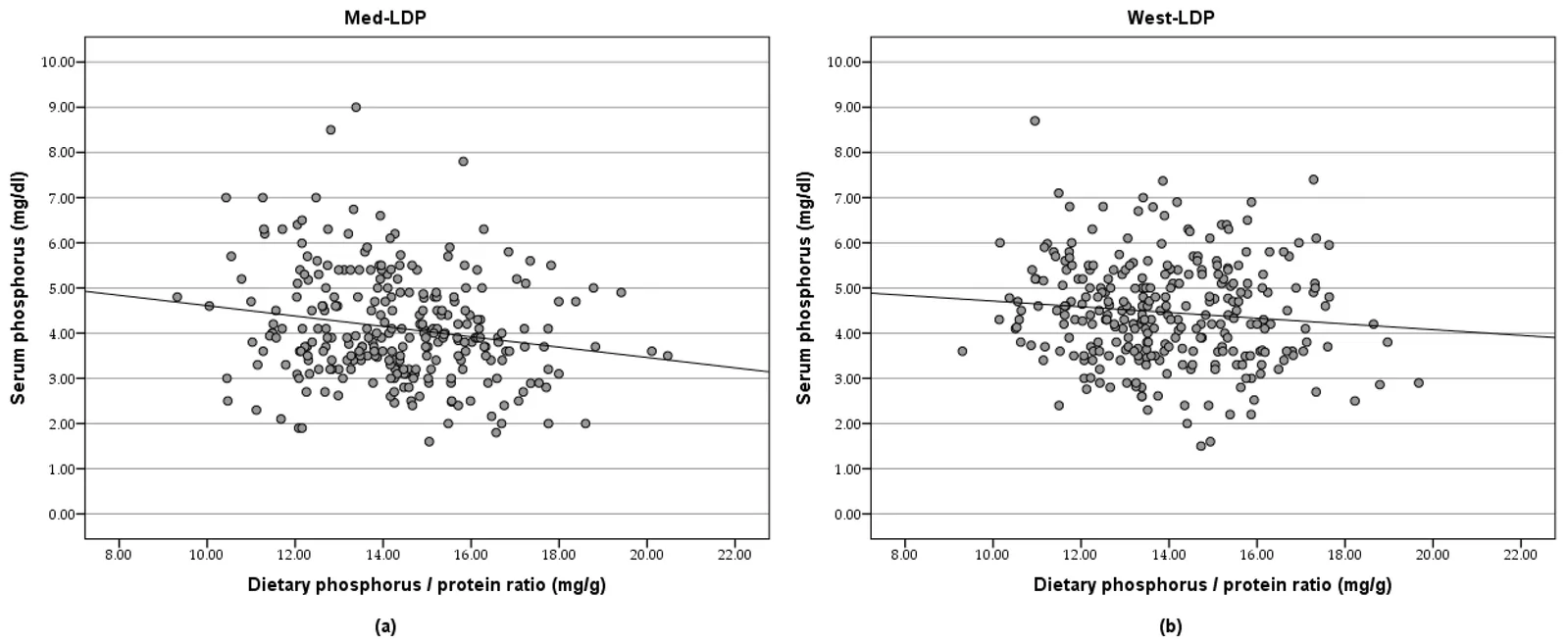
Correlation between serum phosphorus and dietary phosphorus/protein ratio, according to study group. (**a**) Med-LDP: ρ = −0.167, *p* = 0.005. (**b**) West-LDP: ρ = −0.091, *p* = 0.120.

Although a positive correlation between dietary phosphorus intake and serum phosphorus was observed in both groups, the correlation was stronger and statistically significant only in the West-LDP group (ρ = 0.171; *p* = 0.004), whereas it did not reach statistical significance in the Med-LDP group (ρ = 0.114; *p* = 0.059) (Figure 2). In contrast, the phosphorus-to-protein ratio was negatively correlated with serum phosphorus levels in both dietary patterns. The correlation was stronger and statistically significant in the Med-LDP group (ρ = –0.167; *p* = 0.005), whereas it was weaker and not statistically significant in the West-LDP group (ρ = –0.091; *p* = 0.120) (Figure 3).

In addition, the West-LDP was an independent predictor of higher serum phosphorus levels (*p* < 0.001). After adjustment for potential confounders, the West-LDP remained a predictor of higher serum phosphorus levels (*p* = 0.027). However, this association was no longer statistically significant after further adjustment for the prescribed doses of phosphorus binders, calcimimetics, and vitamin D supplementation and analogs (*p* = 0.060) (Table 5).

## 4. Discussion

In this study, no association between phosphorus intake and serum phosphorus levels was found. In addition, two distinct dietary patterns characterizing this population were identified—the Med-LDP (48.6%) and the West-LDP (51.4%)—and the West-LDP was found to be associated with significantly higher serum phosphorus levels, independent of total phosphorus intake. In contrast, the Med-LDP, despite being richer in total phosphorus and protein, was linked to lower serum phosphorus and PTHi levels and a lower calcium–phosphorus product.

Patients following the Med-LDP tended to be older, which may reflect a stronger cultural influence of this diet among older generations, as well as greater health awareness or adherence to dietary recommendations. In contrast, the West-LDP was associated with younger age, which may reflect poorer dietary habits influenced by the dietary westernization, characterized by a higher consumption of inexpensive and ultra-processed foods rich in phosphate additives.

A higher prevalence of DM, as well as a higher age-adjusted comorbidity score was observed in the Med-LDP group. One possible explanation is that patients diagnosed with DM or other clinical conditions (such as cardiovascular diseases, dementia, cancer, etc.) [22] may be more likely to receive dietary counseling and therefore adopt healthier eating habits, including a mediterranean diet. This dietary pattern is widely promoted because of its potential benefits for glycemic control and cardiovascular health [23,24,25,26].

Regarding dietary intake, individuals following the Med-LDP demonstrated higher intakes of energy, protein, dietary fiber, and multiple micronutrients, including phosphorus and potassium. Despite these higher intakes, they exhibited lower serum phosphorus and iPTH level and lower calcium–phosphorus product, while no differences were found in serum potassium, calcium, or sodium levels. These findings suggest that patients following the Med-LDP had a more favorable biochemical profile despite their higher phosphorus and protein intake. This interpretation is further supported by Carrero J. et al. and Garagarza C. et al. [21,27], who observed that patients following a healthy plant-based diet did not have higher serum potassium levels and that greater adherence to the DASH diet was associated with lower serum potassium levels, despite the diet’s naturally higher potassium content. This highlights the importance of the overall dietary quality rather than focusing solely on the absolute intake of single nutrients. However, our observations may also be influenced by differences in baseline clinical characteristics between the groups, including age, comorbidity burden, nutritional status, and residual kidney function. A higher proportion of patients following the Med-LDP had a residual urine output > 200 mL/day, which may have contributed to lower serum phosphorus through preserved residual phosphorus excretion. Nevertheless, residual urine output was included as a confounding factor in the multivariable analysis, and adherence to the Western-like dietary pattern remained independently associated with higher serum phosphorus levels.

In the context of dialysis, protein and energy intake should also be considered, as nutritional adequacy is essential to prevent protein–energy wasting and promote favorable outcomes. In our study, patients following the Med-LDP had higher energy and protein intakes than those adhering to a West-LDP. However, it is worth emphasizing that these intakes remained within the KDOQI-recommended ranges for energy and protein per kilogram of body weight [5]. Importantly, despite the lower nPCR and serum albumin levels observed in the Med-LDP group, both remained within the clinically recommended ranges, supporting the nutritional adequacy and safety of this dietary pattern in patients undergoing HD. Furthermore, no differences in dialysis access or adequacy were observed, suggesting that these factors—known to influence several serum biomarkers, including serum phosphorus—were unlikely to explain the lack of a positive association with phosphorus intake.

The phosphorus-to-protein ratio was inversely associated with serum phosphorus levels in both dietary groups, with a stronger and statistically significant correlation observed in the Med-LDP (ρ = −0.167; *p* = 0.005). In contrast, the association was weaker and not statistically significant in the West-LDP group (ρ = −0.091; *p* = 0.120). Noori et al. reported that higher dietary phosphorus-to-protein ratios were associated with increased mortality risk in patients receiving maintenance HD, supporting the clinical rationale for selecting protein sources with a lower phosphorus burden per gram of protein [28,29]. However, the phosphorus-to-protein ratio does not account for differences in phosphorus bioavailability between food sources. While a lower ratio is generally considered advantageous because it identifies foods that provide less phosphorus per gram of protein, this concept assumes comparable intestinal phosphorus absorption regardless of the food source. In the Mediterranean diet, however, a substantial proportion of phosphorus is derived from plant-based foods such as legumes, nuts, and whole grains, in which phosphorus is largely bound to phytates and therefore less available for intestinal absorption. Consequently, higher phosphorus-to-protein ratios within this dietary pattern may not necessarily result in a greater absorbed phosphorus load, potentially explaining the inverse association observed.

Interestingly, despite the higher total phosphorus intake in the Med-LDP group, serum phosphorus levels remained lower, and no significant association was observed between dietary phosphorus intake and serum phosphorus concentrations. This finding is consistent with that of Noori et al., who also reported a weak correlation between phosphorus intake and serum phosphorus levels [28]. Conversely, a significant positive correlation between dietary phosphorus intake and serum phosphorus levels was observed in the West-LDP (ρ = 0.171, *p* = 0.004). Taken together, these findings suggest that the relationship between dietary phosphorus intake and serum phosphorus levels may depend not only on the quantity consumed but also on the predominant food sources of dietary phosphorus.

The contrasting associations observed between the dietary patterns further support the concept that the clinical interpretation of the phosphorus-to-protein ratio should consider the overall dietary pattern. Since dietary patterns capture the combined effects of multiple foods and nutrients rather than isolated dietary components, the inverse correlation observed in the Med-LDP group may be related to the overall dietary matrix.

As emphasized by St-Jules et al. and Byrne et al., dietary phosphorus management in CKD should consider not only the phosphorus-to-protein ratio but also the phosphorus source, bioavailability, phosphate additives, and the broader food matrix [30,31]. This is particularly relevant to Western dietary patterns, in which processed and ultra-processed foods contribute substantially to phosphorus intake through highly bioavailable phosphate additives. In fact, phosphate additive intake potentially accounts for between 10% and 50% of total phosphorus intake. These additives are widely used in processed and ultra-processed foods, including processed meats, fast food, bakery products, and soft drinks, particularly cola beverages, thereby increasing the intake of highly bioavailable phosphorus, which may not be fully captured by conventional dietary assessment methods [6,29].

The West-LDP was also found to be an independent predictor of higher serum phosphorus levels. Overall, these findings suggest that dietary patterns may be a more relevant determinant of serum phosphorus levels than either total phosphorus intake or the phosphorus-to-protein ratio considered in isolation This association may be partly explained by differences in food sources and phosphorus bioavailability. This challenges the traditional phosphorus-restriction model, which often discourages the consumption of nutrient-dense, phosphorus-rich foods such as legumes and whole grains, and instead supports a more nuanced approach that prioritizes food quality, bioavailability, and the overall dietary context. Accordingly, current guidelines emphasize the individualization of dietary management rather than rigid phosphorus restriction [5]. However, this predictive effect was lost when the model was adjusted for the prescribed dose of phosphorus binders, calcimimetics, and vitamin D supplements and analogs. This may reflect a greater influence of these agents on control serum phosphorus in the context of diets with a high phosphorus content.

Nevertheless, from a clinical perspective, dietary counseling, as one of the cornerstone approaches to control hyperphosphatemia and CKD-MBD in HD patients [2], should extend beyond phosphorus counting and focus on educating patients about phosphorus sources and dietary patterns that may support mineral balance while maintaining nutritional adequacy, as previously demonstrated by Fornasari et al. and Sullivan et al. [32,33]. Further studies are warranted to examine the long-term effects of phosphorus quality and the phosphorus-to-protein ratios on mineral metabolism and health outcomes.

The cross-sectional design of this study can be considered as a limitation because it does not allow causal relationships to be established. The use of a food frequency questionnaire is subject to recall bias and may lead to errors in the estimation of dietary intake. The dietary patterns identified in this study were empirically derived using PCA in a Portuguese cohort and do not represent validated measures of adherence to the Mediterranean or Western diets. Furthermore, the phosphorus content of foods, especially processed foods, can be highly variable and is not consistently documented in nutrient databases, potentially leading to the underestimation or overestimation of intake.

The strengths of this study include its large multicenter sample comprising 564 HD patients from 37 dialysis units which increased the statistical power and supports the generalizability of the findings. Furthermore, the study simultaneously evaluated dietary intake, dietary patterns, mineral metabolism biomarkers, nutritional parameters, and dialysis-related variables, allowing for a comprehensive assessment of phosphorus homeostasis. This included the evaluation of total phosphorus intake, the phosphorus-to-protein ratio, and dietary patterns, providing a broader assessment of dietary phosphorus than would be achieved by assessing isolated nutrients alone. By adopting a dietary pattern approach that reflects real-world eating behaviors, this study provides clinically relevant insights that are aligned with current guideline recommendations and may enhance the translational value of the findings in routine clinical practice.

## 5. Conclusions

In patients undergoing HD, a Mediterranean-like dietary pattern was associated with lower serum phosphorus concentrations despite a higher phosphorus intake. Additionally, this dietary pattern was not linked to increased serum potassium, and patients following this dietary pattern were able to meet the recommended protein and energy intake targets. In contrast, the Western-like dietary pattern was associated with higher serum phosphorus concentrations, although this association was no longer statistically significant after adjustment for pharmacological treatments that may impact serum phosphorus levels.

These findings support individualized dietary counseling that emphasizes phosphorus quality and overall dietary patterns rather than phosphorus restriction alone, while preserving nutritional adequacy. Further longitudinal studies are needed to evaluate the long-term impact of dietary patterns, plant-based protein intake and phosphorus bioavailability on mineral metabolism and clinical outcomes in HD patients.

Future longitudinal or interventional studies should assess the causal relationships between dietary patterns and phosphorus metabolism, considering not only phosphorus intake but also its absorption, excretion, and the use of phosphate binders. Additionally, research should explore the socioeconomic, behavioral, and educational factors that influence the adoption of healthier dietary patterns. More precise measurement tools, including phosphorus bioavailability indices and biomarkers of dietary intake, could further refine our understanding of the relationship between diet and phosphorus metabolism in patients with CKD.

## Figures and Tables

**Table 1:** Factor loadings for two dietary patterns derived by Principal Components Analysis. Data expressed as factor loading (correlation coefficient between each food group and dietary pattern). Food groups with factor loadings ≥ 0.3 were bolded to indicate the main food groups in each component.

Food Groups	Components	
	Med-LDP	West-LDP
Dairy products	0.236	0.008
White meat	0.277	0.206
Red meat	0.224	**0.538**
Olives and olive oil	**0.399**	**0.328**
Oils	0.144	0.241
White bread, rice, pasta and potatoes	**0.316**	0.136
Eggs	**0.353**	0.164
Fried and savory snacks	0.260	**0.555**
Bakery products	**0.328**	0.272
Alcoholic beverages	**0.388**	0.064
Caffeinated drinks	0.062	**0.358**
Fast food	0.017	**0.686**
Vegetable soup	0.237	−0.261
Chicken soup and Portuguese green soup	0.188	−0.094
Barley drinks	0.221	−0.171
Vegetables	**0.649**	0.112
Fruit	**0.630**	−0.001
Whole grain bread	0.051	−0.126
Soft drinks	−0.060	**0.697**
Legumes	**0.550**	0.100
Nuts	0.042	0.151
Fish	**0.578**	0.016

**Table 2:** Food consumption according to the dietary pattern.

Characteristics	Med-LDP (*n* = 274)	West-LDP (*n* = 290)	Effect Size (95% CI)	*p*
Dairy products (g/day) ^1^	250 ± 229	174 ± 176	0.375 (0.208–0.541) ^2^	<0.001
White meat (g/day) ^1^	38 ± 36	33 ± 30	0.149 (−0.016–0.314) ^2^	0.077
Red meat (g/day) ^1^	55 ± 68	71 ± 188	−0.115 (−0.279–0.051) ^2^	0.165
Fish (g/day) ^1^	69 ± 46	41 ± 29	0.712 (0.541–0.881) ^2^	<0.001
Eggs (g/day) ^1^	22 ± 23	15 ± 17	0.333 (0.167–0.499) ^2^	<0.001
Olives and olive oil (g/day) ^1^	14 ± 15	10 ± 13	0.242 (0.076–0.407) ^2^	0.004
Oils (g/day) ^1^	5 ± 6	6 ± 7	−0.084 (−0.249–0.081) ^2^	0.318
White bread, rice, pasta and potatoes (g/day) ^1^	278 ± 190	235 ± 132	0.260 (0.094–0.426) ^2^	0.002
Whole grain bread (g/day) ^1^	86 ± 114	61 ± 88	0.244 (0.078–0.409) ^2^	0.004
Vegetables (g/day) ^1^	109 ± 99	61 ± 59	0.589 (0.420–0.757) ^2^	<0.001
Fruit (g/day) ^1^	314 ± 217	168 ± 107	0.850 (0.677–1.022) ^2^	<0.001
Legumes (g/day) ^1^	22 ± 30	12 ± 16	0.431 (0.264–0.598) ^2^	<0.001
Nuts (g/day) ^1^	2 ± 6	3 ± 1	−0.074 (−0.239–0.091) ^2^	0.380
Vegetable soup (mL/day) ^1^	150 ± 166	48 ± 69	0.810 (0.638–0.981) ^2^	<0.001
Chicken soup and Portuguese green soup (mL/day) ^1^	8 ± 26	2 ± 10	0.318 (0.152–0.484) ^2^	<0.001
Fried and savory snacks (g/day) ^1^	11 ± 15	13 ± 18	−0.102 (−267–0.063) ^2^	0.224
Bakery products (g/day) ^1^	56 ± 72	52 ± 52	0.062 (−0.103–0.227) ^2^	0.459
Alcoholic beverages (mL/day) ^1^	95 ± 155	52 ± 116	0.315 (0.149–0.480) ^2^	<0.001
Caffeinated drinks (mL/day) ^1^	87 ± 83	107 ± 113	−0.196 (−0.361–(−0.0311)) ^2^	0.020
Barley drinks (mL/day) ^1^	47 ± 116	5 ± 25	0.511 (0.343–0.679) ^2^	<0.001
Soft drinks (mL/day) ^1^	18 ± 41	70 ± 121	−0.575 (−0.743–(−0.406)) ^2^	<0.001
Fast Food (g/day) ^1^	2 ± 4	7 ± 14	−0.442 (−0.609–(−0.275)) ^2^	<0.001

^1^ Mean ± Standard Deviation; ^2^ Hodges–Lehmann differences and 95% CI.

**Table 3:** Patients’ characteristics according to the dietary pattern.

Characteristics	Overall (*n* = 564)	Med-LDP (*n* = 274)	West-LDP (*n* = 290)	Effect Size (95% CI)	*p*
**Demographics**					
Age (years) ^2^	71 (61–78)	73 (65–80)	69 (54–76)	5 (3–7) ^3^	<0.001
Male (%)	233 (41.3%)	107 (39.1%)	126 (43.4%)	0.834 (0.596–1.167) ^4^	0.289
Diabetes mellitus (%)	177 (31.4%)	100 (36.5%)	77 (26.6%)	1.590 (1.111–2.275) ^4^	0.011
Age-Adjusted Charlson Index (score)	6 (4–7)	6 (5–8)	5 (4–7)	1 (1–1) ^3^	<0.001
**Dialysis characteristics**					
Hemodialysis vintage (months) ^2^	65 (43–104.8)	62 (41–99)	69 (43–112)	−6 (−12–0) ^3^	0.034
Arteriovenous fistula (%)	468 (83.0%)	229 (83.6%)	239 (82.4%)	0.058 (0.003–0.183) ^5^	0.392
Catheter (%)	36 (6.4%)	20 (7.3%)	16 (5.5%)		
Arteriovenous grafts (%)	60 (10.6%)	25 (9.1%)	35 (12.1%)		
Kt/V—dialysis adequacy ^2^	1.70 (1.50–1.90)	1.70 (1.49–1.92)	1.71 (1.52–1.87)	0.01(−0.06–0.07) ^3^	0.884
OH/ECW pre (%) ^2^	7.3 (2.7–11.88)	7.5 (3.2–11.9)	7.1 (2.2–11.7)	0.7 (−0.6–1.9) ^3^	0.317
Intracellular water (L) ^1^	17.37 ± 3.79	16.99 ± 3.62	17.72 ± 3.91	−0192 (−0.366–(−0.018)) ^6^	0.031
Extracellular water (L) ^2^	16.3 (14.5–18.3)	16.3 (14.6–18.6)	16.0 (14.4–18.2)	−0.2 (−0.3–0) ^3^	0.321
Intradialytic weight gain (%) ^2^	3.1 (2.3–4.0)	2.9 (2.2–4.0)	3.3 (2.3–4.2)	−0.2 (−0.4–0) ^3^	0.079
Residual diuresis > 200 mL (%)	333 (59.3%)	175 (64.3%)	158 (54.5%)	1.507 (1.074–2.116) ^4^	0.017
**Body Composition**					
BMI (kg/m^2^) ^2^	25.8 (22.6–29.0)	26.0 (23.2–29.8)	25.4 (22.3–28.8)	0.71 (−0.08–1.5) ^3^	0.079
Lean Tissue Mass (%) ^2^	46.9 (38.7–56.1)	45.4 (37.6–52.9)	49.3 (40.8–60.1)	−4.2 (−6.6–(−1.9)) ^3^	<0.001
Lean Tissue Index (kg/m^2^) ^2^	12.2 (10.7–14.2)	12 (10.4–13.5)	12.6 (10.9–14.5)	−0.7 (−1.1–(−0.2)) ^3^	0.005
Body Cell Mass Index (kg/m^2^) ^2^	10.7 (8.8–13.2)	10.2 (8.3–12.4)	11.2 (9.0–13.8)	−0.831 (−1.421–(−0.257)) ^3^	0.005
Fat Mass (%) ^2^	36.8 (29.8–42.9)	37.6 (32.9–43.5)	35.2 (27.5–41.6)	2.8 (1.1–4.6) ^3^	0.001
Fat Tissue Index (kg/m^2^) ^2^	13.0 (9.4–16.9)	13.9 (10.7–17.2)	12.2 (8.4–16.3)	1.5 (0.5–2.4) ^3^	0.003
**Biochemical Parameters**					
nPCR (g/kg/day) ^2^	1.1 (1.0–1.3)	1.1 (1.0–1.3)	1.2 (1.1–1.3)	−0.07 (−0.120–(−0.020)) ^3^	0.008
Sodium (mEq/L) ^2^	139 (138–141)	139 (137–141)	140 (138–141)	0 (−1.0–0) ^3^	0.061
Potassium (mEq/L) ^1^	5.3 ± 0.7	5.3 ± 0.7	5.3 ± 0.7	−0.025 (−0.193–0.143) ^6^	0.948
Phosphorus (mg/dL) ^2^	4.2 (3.5–5.0)	3.9 (3.3–4.8)	4.4 (3.6–5.2)	−0.4 (−0.6–(−0.2)) ^3^	<0.001
Calcium (mg/dL) ^2^	8.9 (8.5–9.4)	8.9 (8.6–9.3)	8.9 (8.5–9.5)	0 (−0.1–0.1) ^3^	0.925
Calcium/Phosphorus product [(mg/dL)^2^] ^2^	37.3 (30.7–46.1)	35.0 (29.2–44.9)	39.4 (32.7–47.1)	−3.43 (−5.24–(−1.62)) ^3^	<0.001
PTHi (ng/L) ^2^	352 (211–573)	297 (183–496)	399 (232–618.5)	−80 (−122–(−38)) ^3^	<0.001
Albumin (g/dL) ^2^	4.05 (3.9–4.2)	4 (3.8–4.2)	4.1 (3.9–4.3)	−0.1 (−0.13–(−0.03)) ^3^	<0.001
Ferritin (µg/L) ^2^	455 (329–611)	455(328–620)	455 (329–602)	1.1 (−35.9–37.8) ^3^	0.957
Hemoglobin (g/dL) ^2^	11.2 (10.6–11.9)	11.2 (10.7–11.9)	11.2 (10.6–11.8)	0.1 (−0.1–0.2) ^3^	0.342
**Phosphorus Binders** **weekly prescription (mg)**					
Calcium acetate/magnesium ^1^	2801 ± 5957	2491 ± 5574	3093 ± 6293	−0.101 (−0.266–0.100) ^6^	0.232
Sevelamer carbonate ^1^	2636 ± 7241	1574 ± 5043	3640 ± 8720	−0.288 (−0.453–(−0.122)) ^6^	<0.001
Sucroferric oxyhydroxide ^1^	183 ± 1274	141 ± 1171	223 ± 1366	0.065 (−0.453–(−0.122)) ^6^	0.442

^1^ Mean ± SD; ^2^ Median (IQR); ^3^ Hedges’ g and 95% CI; ^4^ Odds Ratio and 95% CI; ^5^ Cramer’s V and 95% CI; ^6^ Hodges–Lehmann differences and 95% CI; TEI—Total Energy Intake.

**Table 4:** Patients’ dietary intake according to the dietary pattern.

Characteristics	Overall (*n* = 564)	Med-LDP (*n* = 274)	West-LDP (*n* = 290)	Effect Size (95% CI)	*p*
Energy (kcal) ^1^	1808 (1449–2298)	2025 (1654–2515)	1618 (1285–2032)	407 (304–509) ^2^	<0.001
Energy (kcal/kg) ^1^	26.4 (20.4–33.5)	29.2 (23.3–36.6)	24.1 (17.1–30.3)	5.45 (3.838–7.060) ^2^	<0.001
Protein (g) ^1^	75.4 (58.6–98.8)	84.4 (68.3–107.8)	68.2 (50.0–87.4)	18.086 (13.528–22.520) ^2^	<0.001
Protein (g/kg) ^1^	1.1 (0.9–1.4)	1.2 (1.0–1.6)	1.0 (0.7–1.3)	0.240 (0.173–0.309) ^2^	<0.001
Carbohydrates (g) ^1^	240 (184–294)	266 (222–331)	213 (169–266)	54.986 (41.552–68.505) ^2^	<0.001
Complex carbohydrates (g) ^1^	74.5 (56.9–104.8)	83.0 (63.7–115.8)	66.6 (51.6–95.1)	15.421 (10.225–20.841) ^2^	<0.001
Sugar (g) ^1^	69.3 (50.1–95.0)	77.3 (62.1–110.1)	60.4 (43.9–83.8)	19.751 (14.616–24.722) ^2^	<0.001
Total fat (g) ^1^	57.1 (44.1–77.6)	63.4 (49.4–81.1)	50.3 (40.1–71.0)	10.609 (6.937–14.308) ^2^	<0.001
Saturated fat (g) ^1^	17.4 (13.2–24.0)	19.0 (14.7–25.0)	15.6 (12.5–23.3)	2.501 (1.271–3.737) ^2^	<0.001
Monounsaturated fat (g) ^1^	24.7 (18.8–34.0)	27.8 (21.6–35.4)	21.7 (17.0–30.4)	4.983 (3.371–6.629) ^2^	<0.001
Polyunsaturated fat (g) ^1^	9.6 (7.2–12.5)	10.6 (8.3–13.4)	8.4 (6.5–11.3)	1.831 (1.231–2.431) ^2^	<0.001
Cholesterol (mg) ^1^	243 (161–332)	280 (199–348)	208 (142–281)	59.724 (40.996–78.497) ^2^	<0.001
Protein (% TEI) ^1^	16.7 (15.3–18.7)	16.8 (15.3–18.7)	16.6 (15.3–18.7)	0.154 (−0.272–0.583) ^2^	0.479
Carbohydrates (% TEI) ^1^	53.1 (47.7–57.0)	53.4 (48.6–57.0)	52.8 (46.7–57.0)	0.400 (−0.792–1.600) ^2^	0.529
Fat (% TEI) ^1^	29.2 (25.7–32.6)	28.5 (25.3–32.0)	30 (26.2–33.8)	−1.328 (−2.232–(−0.434)) ^2^	0.003
Dietary fiber (g) ^1^	19.1 (14.4–25.9)	23.2 (18.1–30.2)	16.0 (12.4–20.9)	7.081 (5.856–8.363) ^2^	<0.001
Caffeine (mg) ^1^	58.4 (25.7–81.3)	38 (16.0–79.8)	78.8 (31.5–85.9)	−9.502 (−16.312–(−2.461)) ^2^	<0.001
Vitamin B6 (mg) ^1^	1.4 (1.0–1.7)	1.5 (1.3–1.9)	1.1 (0.9–1.5)	0.416 (0.339–0.495) ^2^	<0.001
Vitamin B12 (µg) ^1^	5.0 (3.3–7.4)	6.0 (3.8–8.1)	4.2 (2.7–6.6)	1.473 (0.997–1.964) ^2^	<0.001
Folate (µg) ^1^	206 (156–275)	239 (191–317)	178 (136–233)	67.744 (51.328–78.667) ^2^	<0.001
Vitamin C (mg) ^1^	65.2 (45.3–98.2)	81.8 (58.7–117.06)	52.5 (36.7–76.4)	29.386 (23.892–35.271) ^2^	<0.001
Vitamin D (µg) ^1^	2.5 (1.8–3.6)	2.9 (2.0–4.2)	2.2 (1.5–3.1)	0.695 (0.489–0.905) ^2^	<0.001
Calcium (mg) ^1^	666 (461–894)	769 (557–1024)	555 (395–784)	196.130 (145.102–249.273) ^2^	<0.001
Iron (mg) ^1^	13.3 (10.7–16.6)	14.7 (12.2–18.8)	12.0 (9.4–15.0)	3.044 (2.277–3.809) ^2^	<0.001
Magnesium (mg) ^1^	243 (191–322)	287 (227–366)	212 (161–268)	78.334 (60.749–90.256) ^2^	<0.001
Phosphorus (mg) ^1^	1068 (814–1385)	1206 (941–1559)	954 (701–1213)	278.921 (211.719–346.389) ^2^	<0.001
Phosphorus/protein ratio (mg/g) ^1^	14.0 (12.7–15.4)	14.3 (12.8–15.6)	13.7 (12.5–15.3)	0.433 (0.102–0.751) ^2^	0.010
Potassium (mg) ^1^	2320 (1791–2946)	2661 (2218–3352)	1970(1481–2462)	762.992 (630.765–892.511) ^2^	<0.001
Selenium (µg) ^1^	91.3 (67.8–139.7)	108.2 (79.1–158.6)	78.8 (62.1–116.4)	26.906 (19.728–34.289) ^2^	<0.001
Sodium (mg) ^1^	2196 (1634–2825)	2456 (1837–2998)	1972 (1464–2534)	448.257 (312.820–583.106) ^2^	<0.001
Zinc (mg) ^1^	8.8 (6.7–11.6)	9.9 (7.6–13.1)	7.9 (5.8–10.4)	2.055 (1.499–2.591) ^2^	<0.001

^1^ Median (IQR); ^2^ Hodges–Lehmann differences and 95% CI; TEI—Total Energy Intake.

**Table 5:** Linear Regression Analysis Between Serum Phosphorus and Dietary Pattern.

Dependent Variable	Independent Variable	B (95% CI) (*n* = 564)	*p*	B ^a^ (95% CI) (*n* = 355)	*p*	B ^b^ (95% CI) (*n* = 352)	*p*
Serum phosphorus	Dietary Pattern (West-LDP; Indicator: Med-LDP)	0.35 (0.16–0.54)	<0.001	0.29 (0.03–0.54)	0.027	0.24 (−0.01–0.49)	0.060

^a^ Adjusted for: age, gender, DM, dialysis vintage, PTHi, dialysis dose (Kt/V), pre-dialysis albumin, energy (kcal), phosphorus/protein ratio, vascular access, residual diuresis > 200 mL. ^b^ Adjusted for: age, gender, DM, dialysis vintage, PTHi, dialysis dose (Kt/V), pre-dialysis albumin, energy (kcal), phosphorus/protein ratio, vascular access, residual diuresis > 200 mL, weekly prescription of phosphorus binders, calcimimetics, and vitamin D supplementation and analogs.

## Data Availability

The data underlying this article cannot be shared publicly due to patient confidentiality and ethical and privacy restrictions.

## Abbreviations

The following abbreviations are used in this manuscript:

HD	Hemodialysis
CKD	Chronic Kidney disease
CKD-MBD	CKD-Mineral Bone Disease
CV	Cardiovascular events
BCM	Body Composition Monitor
LTI	Lean Tissue Index
FTI	Fat Tissue Index
BCMI	Body Cell Mass Index
OH/ECW	Overhydration/extracellular water pre-dialysis
Ca/P	Calcium/phosphorus product
PTHi	Intact parathyroid hormone
nPCR	normalized Protein Catabolic Rate
PCA	Principal Component Analysis
SD	Standard Deviation
IQR	Interquartile range
DM	Diabetes Mellitus
Med-LDP	Mediterranean-like dietary pattern
West-LDP	Western-like dietary pattern

## References

[1] Blayney M.J., Tentori F. (2009). Trends and Consequences of Mineral Bone Disorder in Haemodialysis Patients: Lessons from the Dialysis Outcomes and Practice Patterns Study (DOPPS). J. Ren. Care.

[2] Rastogi A., Bhatt N., Rossetti S., Beto J. (2021). Management of Hyperphosphatemia in End-Stage Renal Disease: A New Paradigm. J. Ren. Nutr..

[3] Yamada S., Nakano T. (2023). Role of Chronic Kidney Disease (CKD)–Mineral and Bone Disorder (MBD) in the Pathogenesis of Cardiovascular Disease in CKD. J. Atheroscler. Thromb..

[4] Palmer S.C., Hayen A., Macaskill P., Pellegrini F., Craig J.C., Elder G.J., Strippoli G.F.M. (2011). Serum Levels of Phosphorus, Parathyroid Hormone, and Calcium and Risks of Death and Cardiovascular Disease in Individuals with Chronic Kidney Disease a Systematic Review and Meta-Analysis. JAMA.

[5] Ikizler T.A., Burrowes J.D., Byham-Gray L.D., Campbell K.L., Carrero J.J., Chan W., Fouque D., Friedman A.N., Ghaddar S., Goldstein-Fuchs D.J. (2020). KDOQI Clinical Practice Guideline for Nutrition in CKD: 2020 Update. Am. J. Kidney Dis..

[6] Vervloet M.G., van Ballegooijen A.J. (2018). Prevention and Treatment of Hyperphosphatemia in Chronic Kidney Disease. Kidney Int..

[7] Garagarza C., Valente A., Caetano C., Ramos I., Sebastião J., Pinto M., Oliveira T., Ferreira A., Guerreiro C.S. (2023). Mediterranean Diet: A Dietary Pattern Related to Nutritional Benefits for Hemodialysis Patients. J. Ren. Nutr..

[8] Dominguez L.J., Veronese N., Baiamonte E., Guarrera M., Parisi A., Ruffolo C., Tagliaferri F., Barbagallo M. (2022). Healthy Aging and Dietary Patterns. Nutrients.

[9] English L.K., Ard J.D., Bailey R.L., Bates M., Bazzano L.A., Boushey C.J., Brown C., Butera G., Callahan E.H., de Jesus J. (2021). Evaluation of Dietary Patterns and All-Cause Mortality A Systematic Review. JAMA Netw. Open.

[10] Obbagy J., Essery E. (2020). Dietary Patterns and Risk of Cardiovascular Disease: A Systematic Review.

[11] Wang W., Liu Y., Li Y., Luo B., Lin Z., Chen K., Liu Y. (2023). Dietary Patterns and Cardiometabolic Health: Clinical Evidence and Mechanism. MedComm.

[12] Ellouze I., Sheffler J., Nagpal R., Arjmandi B. (2023). Dietary Patterns and Alzheimer’s Disease: An Updated Review Linking Nutrition to Neuroscience. Nutrients.

[13] Booth S., Talegawkar S., Fung T., Hoelscher D.M., Anderson C.A.M., Deierlein A., Gardner C., Giovannucci E., Raynor H., Stanford F.C. (2024). Dietary Patterns and Risk of Cognitive Decline, Dementia, Alzheimer’s Disease, and Mild Cognitive Impairment: A Systematic Review.

[14] Marcelli D., Usvyat L.A., Kotanko P., Bayh I., Canaud B., Etter M., Gatti E., Grassmann A., Wang Y., Marelli C. (2015). Body Composition and Survival in Dialysis Patients: Results from an International Cohort Study. Clin. J. Am. Soc. Nephrol..

[15] Duarte M.P., Martins P., Leal D.V., Nóbrega O.T., Ribeiro H.S., Viana J.L. (2025). Association of Diabetes with Sarcopenia in Patients on Hemodialysis: A Nationwide Cross-Sectional Study in Portugal. Clin. Nutr. ESPEN.

[16] Wabel P., Chamney P., Moissl U., Jirka T. (2009). Importance of Whole-Body Bioimpedance Spectroscopy for the Management of Fluid Balance. Blood Purif..

[17] Onofriescu M., Siriopol D., Voroneanu L., Hogas S., Nistor I., Apetrii M., Florea L., Veisa G., Mititiuc I., Kanbay M. (2015). Overhydration, Cardiac Function and Survival in Hemodialysis Patients. PLoS ONE.

[18] Lopes C.M.D.M. (2000). Reprodutibilidade e Validação de Um Questionário Semiquantitativo de Frequência Alimentar. Alimentação e Enfarte Agudo Do Miocárdio; Estudo Caso-Controlo de Base Comunitária.

[19] Lopes C., Aro A., Azevedo A., Ramos E., Barros H. (2007). Intake and Adipose Tissue Composition of Fatty Acids and Risk of Myocardial Infarction in a Male Portuguese Community Sample. J. Am. Diet. Assoc..

[20] Ferreira F., Graça M., Instituto Nacional de Saúde Dr. Ricardo Jorge (INSA), Editorial do Ministério da Educação (1985). Tabela de Composição de Alimentos Portugueses.

[21] Garagarza C., Valente A., Caetano C., Ramos I., Sebastião J., Pinto M., Oliveira T., Ferreira A., Guerreiro C.S. (2022). Potassium Intake—(Un)Expected Non-Predictor of Higher Serum Potassium Levels in Hemodialysis Dash Diet Consumers. Nutrients.

[22] Zhou S., Zhang X.H., Zhang Y., Gong G., Yang X., Wan W.H. (2022). The Age-Adjusted Charlson Comorbidity Index Predicts Prognosis in Elderly Cancer Patients. Cancer Manag. Res..

[23] Wu M.J., Hung C.H., Yong S.B., Ching G.S., Hsu H.J. (2025). Impact of the Mediterranean Diet on Glycemic Control, Body Mass Index, Lipid Profile, and Blood Pressure in Type 2 Diabetes: A Meta-Analysis of Randomized Controlled Trials. Nutrients.

[24] Esposito K., Maiorino M.I., Bellastella G., Chiodini P., Panagiotakos D., Giugliano D. (2015). A Journey into a Mediterranean Diet and Type 2 Diabetes: A Systematic Review with Meta-Analyses. BMJ Open.

[25] Salas-Salvadó J., Díaz-López A., Ruiz-Canela M., Basora J., Fitó M., Corella D., Serra-Majem L., Wärnberg J., Romaguera D., Estruch R. (2019). Effect of a Lifestyle Intervention Program With Energy-Restricted Mediterranean Diet and Exercise on Weight Loss and Cardiovascular Risk Factors: One-Year Results of the PREDIMED-Plus Trial. Diabetes Care.

[26] Jing T., Zhang S., Bai M., Chen Z., Gao S., Li S., Zhang J. (2023). Effect of Dietary Approaches on Glycemic Control in Patients with Type 2 Diabetes: A Systematic Review with Network Meta-Analysis of Randomized Trials. Nutrients.

[27] Carrero J.J., González-Ortiz A., Avesani C.M., Bakker S.J.L., Bellizzi V., Chauveau P., Clase C.M., Cupisti A., Espinosa-Cuevas A., Molina P. (2020). Plant-Based Diets to Manage the Risks and Complications of Chronic Kidney Disease. Nat. Rev. Nephrol..

[28] Noori N., Kalantar-Zadeh K., Kovesdy C.P., Bross R., Benner D., Kopple J.D. (2010). Association of Dietary Phosphorus Intake and Phosphorus to Protein Ratio with Mortality in Hemodialysis Patients. Clin. J. Am. Soc. Nephrol..

[29] Watanabe M.T., Araujo R.M., Vogt B.P., Barretti P., Caramori J.C.T. (2016). Most Consumed Processed Foods by Patients on Hemodialysis: Alert for Phosphate-Containing Additives and the Phosphate-to-Protein Ratio. Clin. Nutr. ESPEN.

[30] Byrne F.N., Gillman B., Kiely M., Bowles M., Connolly P., Earlie J., Murphy J., Rennick T., Reilly E.O., Shiely F. (2021). Revising Dietary Phosphorus Advice in Chronic Kidney Disease G3-5D. J. Ren. Nutr..

[31] St-Jules D.E., Woolf K., Pompeii M.-L., Kalantar-Zadeh K., Sevick M.A. (2016). Re-Examining the Phosphorus-Protein Dilemma: Does Phosphorus Restriction Compromise Protein Status?. J. Ren. Nutr..

[32] de Fornasari M.L.L., dos Santos Sens Y.A. (2017). Replacing Phosphorus-Containing Food Additives with Foods Without Additives Reduces Phosphatemia in End-Stage Renal Disease Patients: A Randomized Clinical Trial. J. Ren. Nutr..

[33] Sullivan C., Sayre S.S., Leon J.B., Machekano R., Love T.E., Porter D., Marbury M., Sehgal A.R. (2009). Effect of Food Additives on Hyperphosphatemia among Patients with End-Stage Renal Disease: A Randomized Controlled Trial. JAMA.

